# Retrospective report of antimicrobial susceptibility observed in bacterial pathogens isolated from ocular samples at Mount Sinai Hospital, 2010 to 2015

**DOI:** 10.1186/s13756-017-0185-0

**Published:** 2017-03-20

**Authors:** Marko Oydanich, Tanis C. Dingle, Camille L. Hamula, Claudia Ghisa, Penny Asbell

**Affiliations:** 1 Department of Ophthalmology, Icahn School of Medicine at Mount Sinai, New York, NY USA; 2 Department of Pathology, Icahn School of Medicine at Mount Sinai, New York, NY 10029 USA

**Keywords:** Antimicrobial, Multi-drug resistance, Ocular isolates, Susceptibility, New York

## Abstract

**Background:**

Antimicrobial resistance has emerged as a major threat to global public health. Thus, the surveillance of changes in antimicrobial resistance in local and global settings is a paramount necessity. While many studies have tracked antimicrobial resistance, only a small percentage surveyed ocular isolates. The purpose of this study was to report the in vitro susceptibility of bacterial pathogens isolated from ocular samples in New York, NY from 2010 to 2015.

**Methods:**

A retrospective review of ocular isolates was conducted. All organisms were collected by 25 separate inpatient wards and outpatient clinics, and were analyzed by the clinical microbiology laboratory at Mount Sinai Hospital. Clinical Laboratory and Standards Institute (CLSI) guidelines were followed for susceptibility testing and breakpoint interpretations.

**Results:**

A total of 549 bacterial organisms were isolated from 1664 cultures (33%) during the 6-year study period. Of these, 358 isolates (65.2%) underwent susceptibility testing. 182 (50.8%) isolates were Gram-positive. The most common Gram-positive bacterium was *Staphylococcus aureus* (62.1%). Methicillin-resistance decreased in *S. aureus* isolates (31.3% in 2010, 14.1% in 2015) but was without significant change (*p* = 0.25). When analyzing all *S. aureus* isolates recovered during the study period, there were significantly more methicillin-resistant *S. aureus* (MRSA) isolates resistant to fluoroquinolones (*p* <0.0001), erythromycin (*p* <0.0001), and trimethoprim/sulfamethoxazole (TMP/SMZ; *p* <0.05). Overall, *Streptococcus pneumoniae* isolates showed reduced susceptibility to erythromycin, but were otherwise susceptible to the other antimicrobials tested. *Haemophilus influenzae* (26.1%) and *Pseudomonas aeruginosa* (23.9%) were the most common Gram-negative bacteria isolated*.* Resistance to ampicillin and TMP/SMZ was observed in several of the *H. influenzae* isolates. *P. aeruginosa* isolates did not show high resistance overall, however, it was noted that isolates resistant to meropenem were also resistant to other antimicrobials (*p* < 0.01).

**Conclusion:**

Overall, antimicrobial resistance was infrequent for the Gram-negative and Gram-positive bacteria analyzed. While the MRSA isolates demonstrated increased resistance to multiple antimicrobial classes, this is expected for this pathogen. Due to the continued use of broad-spectrum oral and systemic antimicrobials to treat ocular infections, findings of this study and other surveillance studies specific to ocular isolates should be used as resources in effective decision making in the treatment of ocular disease.

## Background

Antimicrobial resistance is an issue that has been impacting medicine for many years. Whether in primary care or specialized care settings, antimicrobial resistance has been on the rise and has affected the way healthcare providers treat bacterial infections. Particularly in ophthalmic settings, physicians tend to treat ocular infections by prescribing empiric antibiotics because they often do not have data from culture analysis. As a result, there is a lack of awareness of fluctuating trends in antimicrobial resistance of many common pathogens [1–4]. This is an important concern, especially due to the fact that many ocular pathogens, if shown to possess multi-drug resistance (MDR), i.e. non-susceptibility to at least one agent in three or more antimicrobial classes [5], can be difficult to treat, which increases the chances of severe ocular damage and vision loss [3, 6, 7].

Generally, broad-spectrum antibiotics are used to treat many ocular infections, including microbial keratitis, conjunctivitis, and endophthalmitis; however, the pathogenic organisms that cause these conditions have shown increased resistance to antibiotics over the past decade. For example, *Staphylococcus* species and *Pseudomonas aeruginosa* have demonstrated resistance to fluoroquinolones, macrolides, and β-lactams [2, 3, 6, 8]. *Haemophilus influenzae*, a common Gram-negative bacterium, has also shown increased resistance to trimethoprim [9–12].

There have been a few nationwide surveillance studies, such as Ocular TRUST and ARMOR, which have tracked resistance patterns in many common ocular pathogens [9, 13]. However, given the growing importance of this issue, there is also a need for smaller, regional studies to obtain local data. Many such single-center studies have been surveying and tracking antimicrobial resistance in order to establish effective treatment methods against ocular infections on a local level [6, 14–19]. The goal of this retrospective study was to determine resistance patterns in ocular pathogens isolated and analyzed by the Mount Sinai Hospital clinical microbiology laboratory in New York, NY and contribute our findings to the growing knowledge base regarding antimicrobial resistance of ocular pathogens. In turn, these findings can also help influence intelligent and effective antibiotic use in the New York City metropolitan area, with the potential to help develop informed treatment protocols against common ocular pathogens.

## Methods

A retrospective, consecutive data review study was conducted. The study was approved by the institutional review board of Mount Sinai Hospital and the tenets of the Declaration of Helsinki were followed.

### Collection of ocular isolates

A total of 1664 ocular cultures were performed from over 25 separate inpatient wards and outpatient clinics over a 6-year period (January 2010 to December 2015). The clinical microbiology laboratory at Mount Sinai Hospital performed all cultures and antimicrobial susceptibility testing for these facilities. For the purposes of this review, only specimens that tested positive for a bacterial organism were included in the study. Fungal isolates were not analyzed.

### Eye culture workup and organism identification

Eye culture specimens (swabs of various ocular anatomic sites, corneal scrapings, and vitreous/aqueous fluids) were either directly plated to agar at the time of collection or sent to the clinical microbiology laboratory for plating. The routine culture media used for eye cultures included blood agar, chocolate agar, and CDC anaerobe 5% sheep blood agar. Providers collecting specimens at the bedside would also frequently add a thioglycollate broth during collection. All media was incubated aerobically in a 5% CO_2_ environment or under anaerobic conditions at 35–37 **°**C per laboratory standard operating protocols. Positive cultures were determined by the laboratory’s standard operating protocol. For specimens that were directly plated (isolated from corneal scrapings or vitreous fluids), the organism had to be localized to the area of the plate where the specimen was planted. For those specimens collected with a swab, the organism had to be localized in the first quadrant (first streak area) of the plate. If growth was observed in the 2^nd^, 3^rd^, or 4^th^ area, consultation with the microbiology director typically resulted in the organism being labeled as a contaminant.

Significant ocular pathogens were primarily identified using routine biochemical testing and the VITEK-2 instrument (bioMérieux, Inc., Durham, NC). If the VITEK-2 result failed, alternate identification systems used by the laboratory included API strips (bioMérieux, Inc., Durham, NC) and the rapID identification system (Remel, Lenexa, KS).

### Susceptibility testing

All susceptibility testing was performed in the Mount Sinai Hospital clinical microbiology laboratory. A total of 358 isolates underwent susceptibility testing from a total of 549 isolated bacterial isolates. Only significant ocular pathogens as defined by the laboratory standard operating protocol underwent susceptibility testing. Organisms identified that were believed to be contaminants or normal flora (i.e. Coagulase-negative *Staphylococcus* and *Corynebacterium* species) were excluded from analysis since susceptibility testing was not performed. This included 152 coagulase-negative *Staphylococcus* (CoNS) isolates that were collected during the 6-year period. Providers always had the opportunity to call the laboratory and request susceptibility testing on these isolates if they deemed them significant. The panel of antimicrobials tested was determined by the microbiology director and antimicrobial stewardship based on the institution’s antibiotic formulary. The primary susceptibility testing method used in the laboratory during the study was VITEK-2 (bioMérieux, Inc., Durham, NC), which was performed per manufacturer’s recommendations. *Streptococcus* species, infrequently isolated, and fastidious organisms were tested by E-test (bioMérieux Inc., Durham, NC) per manufacturer’s recommendations. Interpretations (susceptible, intermediate, susceptible dose-dependent, or resistant) were determined using Clinical Laboratories and Standards Institute (CLSI) guidelines (M100 [20] or M45 [21] documents) relevant to the year the organism was tested.

### Data organization & inclusion procedures

All data was collected from the Laboratory Information System used by the Mount Sinai Hospital clinical microbiology laboratory. The data was imported into a Microsoft Excel spreadsheet file and all important patient identifiers were properly and securely discarded in accordance with IRB guidelines. The data imported included date of collection, age of patient, ward, collection site, organism isolated and MIC values against a variety of antibiotics. Only isolates that underwent susceptibility testing were included in this study. Susceptibility profiles are shown for microbial species that were tested at least 30 times during the study period with the exception of MRSA (*n* = 28), though not all antimicrobials were tested against each isolate. MIC_50_ and MIC_90_ values were also calculated.

### Statistical analysis

Statistical analysis was conducted using the program Prism (software version 7.0a; GraphPad software). A Fisher’s exact test was used to study the prevalence of methicillin-resistance amongst all *Staphylococcus aureus* isolates during the study period as well as to compare differences in resistance between methicillin-resistant *Staphylococcus aureus* (MRSA) and methicillin-susceptible *Staphylococcus aureus* (MSSA) isolates. Comparisons amongst *S. aureus* and age of infection were conducted using a Chi-Squared test. Statistical significance was defined as *p* < 0.05.

## Results

Of a total of 1664 cultures performed during the study period, 549 bacterial organisms were isolated. Of these, 358 ocular pathogens (65.2%) underwent susceptibility testing, which included 113 *S. aureus*, 30 *S. pneumoniae,* 42 *P. aeruginosa*, & 46 *H. influenzae* isolates (Table 1). Of the 358 isolates, 41 (11.5%), 30 (8.4%), 11 (3.1%), 17 (4.7%), and 3 (0.8%) samples were isolated from the conjunctiva, cornea, eyelid, contact lens, and vitreous fluid respectively. The remaining 256 (71.5%) samples were collected from unspecified ocular sites. Gram-positive bacteria accounted for 50.8% of the total isolates. *S. aureus* was the most common Gram-positive bacterium [113 of 182 (62.1%)] while *H. influenzae* was the most common Gram-negative bacterium [46 of 176 (26.1%)]. Comparison by patient age, in years, (Table 2) shows that the amount of Gram-positive organisms (*S. aureus*, coagulase-negative *Staphylococcus* (CoNS), and *S. pneumoniae* isolates) was higher in the elderly group (>65 years) than in any other group and increased in an age-dependent manner. MRSA infections were statistically more likely to occur in the two oldest age groups, i.e. >40 years, when compared to the younger age groups (p < 0.05). Gram-negative organisms, with the exception of *H. influenzae,* were more prevalent in the middle-aged groups, i.e. 18–64 years. Interestingly, *H. influenzae* isolates were more prevalent in the youngest and oldest age groups, presenting a bimodal distribution.

**Table 1:** Ocular Isolates Collected & Analyzed at Mount Sinai Hospital (2010–2015)

Gram-Positive Organisms	No.	%Gram-Positive	%Total	Gram-Negative Organisms	No.	%Gram-Negative	%Total
***Staphylococcus aureus***				**Non-Fermenting Gram-Negative Bacilli**			
**MSSA**	85	46.70%	23.74%	***Pseudomonas aeruginosa***	42	23.86%	11.73%
**MRSA**	28	15.38%	7.82%	*Stenotrophomonas maltophilia*	7	3.98%	1.96%
**CoNS**				*Achromobacter xylosoxidans*	6	3.41%	1.68%
*Staphylococcus epidermidis*	13	7.14%	3.63%	*Moraxella catarrhalis*	4	2.27%	1.12%
*Staphylococcus haemolyticus*	2	1.10%	0.56%	*Acinetobacter baumannii*	3	1.70%	0.84%
*Staphylococcus warneri*	2	1.10%	0.56%	*Pseudomonas luteola*	1	0.57%	0.28%
*Staphylococcus capitis*	1	0.55%	0.28%	*Pseudomonas putida*	1	0.57%	0.28%
*Unspecified CoNS*	2	1.10%	0.56%	*Pseudomonas (Flavimonas) oryzihabitans*	1	0.57%	0.28%
**Other**				*Unspecified Acinetobacter spp.*	1	0.57%	0.28%
***Streptococcus pneumoniae***	30	16.48%	8.38%	*Moraxella lacunata*	1	0.57%	0.28%
*Corynebacterium pseudodiphthericum*	3	1.65%	0.84%	*Unspecified Moraxella spp.*	1	0.57%	0.28%
*Streptococcus pyogenes (Group A)*	2	1.10%	0.56%	*Achromobacter (Alcaligenes) denitrificans*	1	0.57%	0.28%
*Streptococcus intermedius*	2	1.10%	0.56%	*Chryseobacterium indologenes*	1	0.57%	0.28%
*Unspecified Corynebacterium spp.*	2	1.10%	0.56%	*Elizabethkingia meningoseptica*	1	0.57%	0.28%
*Bacillus cereus*	2	1.10%	0.56%	**Enterobacteriaceae**			
*Streptococcus anginosus*	1	0.55%	0.28%	*Serratia marcescens*	18	10.23%	5.03%
*Streptococcus mitis*	1	0.55%	0.28%	*Escherichia coli*	10	5.68%	2.79%
*Enterococcus faecium*	1	0.55%	0.28%	*Klebsiella oxytoca*	7	3.98%	1.96%
*Enterococcus faecalis*	1	0.55%	0.28%	*Klebsiella pneumoniae*	5	2.84%	1.40%
*Corynebacterium (CDC Group G)*	1	0.55%	0.28%	*Enterobacter cloacae*	2	1.14%	0.56%
*Gemella morbillorum*	1	0.55%	0.28%	*Citrobacter koseri*	2	1.14%	0.56%
*Unspecified Bacillus spp.*	1	0.55%	0.28%	*Pantoea agglomerans*	2	1.14%	0.56%
*Aerococcus viridans*	1	0.55%	0.28%	*Raoultella ornithinolytica*	1	0.57%	0.28%
				*Citrobacter freundii*	1	0.57%	0.28%
				*Providencia rettgeri*	1	0.57%	0.28%
				*Morganella morganii*	1	0.57%	0.28%
				*Proteus mirabilis*	1	0.57%	0.28%
				**Other**			
				***Haemophilus influenzae***	46	26.14%	12.85%
				*Haemophilus parainfluenzae*	2	1.14%	0.56%
				*Aggregatibacter aphrophilus*	2	1.14%	0.56%
				*Eikenella corrodens*	2	1.14%	0.56%
				*Neisseria meningitidis*	1	0.57%	0.28%
				*Pasteurella multocida*	1	0.57%	0.28%

Bolded organisms are commonly observed bacterial pathogens

Bolded and underlined terms represent important classes of microorganisms

*Abbreviation: MSSA* methicillin-susceptible *S. aureus*, *MRSA* methicillin-resistant *S. aureus, CoNS* coagulase-negative *Staphylococcus, spp.* species

**Table 2:** Frequency of ocular pathogens by age group

Microorganism	Age Distribution (years)				
	≤2	3–17	18–39	40–64	≥65
	*N* (%)	*N* (%)	*N* (%)	*N* (%)	*N* (%)
MSSA	14 (16.5%)	8 (9.4%)	15 (18.8%)	18 (21.2%)	30 (35.3%)
MRSA	2 (7.1%)	2 (7.1%)	2 (7.1%)	11 (39.3%)	11 (39.3%)
CoNS	2 (10.0%)	1 (5.0%)	1 (5.0%)	6 (30.0%)	10 (50.0%)
*Streptococcus pneumoniae*	5 (16.7%)	2 (6.7%)	2 (6.7%)	12 (40.0%)	9 (30.0%)
*Pseudomonas aeruginosa*	1 (2.4%)	3 (7.1%)	12 (28.6%)	17 (40.5%)	9 (21.4%)
*Haemophilus influenzae*	14 (30.4%)	5 (10.9%)	3 (6.5%)	12 (26.1%)	12 (26.1%)
*Klebsiella spp.*	4 (33.3%)	0 (0%)	3 (25%)	2 (16.7%)	3 (25%)
*Serratia marcescens*	1 (5.6%)	1 (5.6%)	8 (44.4%)	6 (33.3%)	2 (11.1%)

*Abbreviations: MSSA* = methicillin-susceptible *Staphylococcus aureus, MRSA* = methicillin-resistant *Staphylococcus aureus, CoNS* = coagulase-negative *Staphylococcus, spp* = species

### Staphylococcus aureus

#### MRSA

**Fig. 1:**
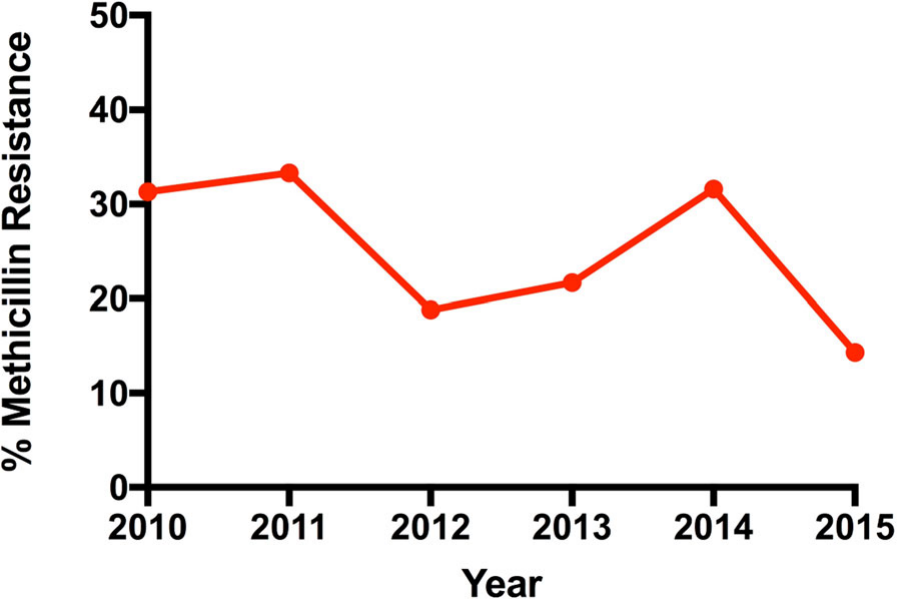
Prevalence of MRSA from 2010–2015. An overall decreasing trend in MRSA prevalence is observed from 2010–2015. However, the trend is not statistically significant (*p* = 0.25).

Over the 6-year period that was evaluated, the prevalence of methicillin-resistant *S. aureus* showed a decreasing yet statistically insignificant trend (Fig. 1), with 31.30% resistance in 2010 compared to 14.30% resistance in isolates collected in 2015 (*p* = .25).

A total of 28 cultures tested positive for methicillin-resistant *S. aureus* (Table 3a). MRSA isolates exhibited high resistance against erythromycin (89.29%), clindamycin (35.7%), levofloxacin (50%), and ciprofloxacin (80%) and high susceptibility to the remainder of the antimicrobials tested. No MRSA isolates showed resistance to vancomycin, daptomycin or linezolid.

**Table 3:** Susceptibility profiles for selected Gram-positive species, *S. aureus* (a) and *S. pneumoniae* (b)

**3a: Susceptibility Profile for 113 S.** ***aureus*** **isolates**							
Antimicrobial		MIC (ug/mL)			Interpretation N (%)		
	Organism	Range	MIC_50_	MIC_90_	Susceptible	Intermediate	Resistant
Clindamycin	MRSA	0.25 – ≥8	0.25	≥8	18 (64.3)	0 (0)	10 (35.7)
	MSSA	≤0.12 – ≥8	0.25	<0.5	56 (65.9)	0 (0)	29 (34.1)
Ciprofloxacin*	MRSA	≤0.5 – ≥8	≥8	≥8	5 (20)	0 (0)	20 (80)
	MSSA	≤0.5 – ≥8	≤0.5	≤0.5	74 (91.4)	1 (1.2)	6 (7.4)
Daptomycin	MRSA	0.25 – 1	0.5	1	28 (100)	0 (0)	0 (0)
	MSSA	≤0.12 – 1	0.25	0.5	85 (100)	0 (0)	0 (0)
Erythromycin	MRSA	≤0.25 – ≥8	≥8	≥8	3 (10.7)	0 (0)	25 (89.3)
	MSSA	≤0.25 – ≥8	0.5	≥8	47 (56)	0 (0)	37 (44)
Gentamicin	MRSA	≤0.5 – ≥16	≤0.5	8	24 (85.7)	2 (7.1)	2 (7.1)
	MSSA	≤0.5 – 4	≤0.5	≤0.5	85 (100)	0 (0)	0 (0)
Levofloxacin	MRSA	0.25 – ≥8	4	≥8	6 (21.4)	8 (28.6)	14 (50)
	MSSA	≤0.12 – ≥8	0.25	2	80 (94.1)	2 (2.4)	3 (3.5)
Linezolid	MRSA	2 – 4	2	2	28 (100)	0 (0)	0 (0)
	MSSA	1 – 4	2	2	85 (100)	0 (0)	0 (0)
Trimethoprim/Sulfamethoxazole	MRSA	≤0.5 – ≥16	≤0.5	8	25 (89.3)	0 (0)	3 (10.7)
	MSSA	≤0.5 – ≥16	0.5	0.5	84 (98.8)	0 (0)	1 (1.2)
Tetracycline*	MRSA	≤1 – ≥16	≤1	4	22 (91.7)	0 (0)	2 (8.3)
	MSSA	≤1 – ≥16	≤1	4	48 (94.1)	0 (0)	3 (5.9)
Vancomycin	MRSA	≤0.5 – 2	1	2	28 (100)	0 (0)	0 (0)
	MSSA	≤0.5 – 2	1	1	37 (100)	0 (0)	0 (0)
**3b: Susceptibility Profile for 30 S.** ***pneumoniae*** **isolates**							
Antimicrobial	MIC (ug/mL)				Interpretation N (%)		
		Range	MIC_50_	MIC_90_	Susceptible	Intermediate	Resistant
Ceftriaxone (non-meningitis)^#^		.004 – 0.5	0.032	0.25	23 (100)	0 (0)	0 (0)
Clindamycin^#^		.032 – 1	0.125	0.25	27(93.2)	1 (3.4)	1 (3.4)
Erythromycin		.016 – 32	0.25	2	22 (73.3)	3 (10)	5 (16.7)
Levofloxacin		0.25 – 4	1	1	29 (96.7)	0 (0)	1 (3.3)
Penicillin (non-meningitis)^#^		.008 – 2	0.032	0.5	25 (100)	0 (0)	0 (0)
Vancomycin		.25 – 2	0.5	1	29 (96.7)	0 (0)	1 (3.3)

*Antimicrobials that were not tested against all 28 MRSA isolates

#Antimicrobials that were not tested against 30 or more *S. pneumoniae* isolates

*Abbreviations: MSSA* = methicillin-susceptible *Staphylococcus aureus, MRSA* = methicillin-resistant *Staphylococcus aureus, MIC* = minimum inhibitory concentration (at 50% and 90%)

#### MSSA

A total of 85 cultures tested positive for methicillin-susceptible *S. aureus* (Table 3a). MSSA isolates exhibited high susceptibility to the majority of antimicrobials tested with the exception of clindamycin (34.12%) and erythromycin (44.05%). No isolates were found to be resistant to vancomycin, daptomycin, gentamicin, or linezolid. Unlike MRSA, MSSA isolates were highly susceptible to levofloxacin and ciprofloxacin.

**Fig. 2:**
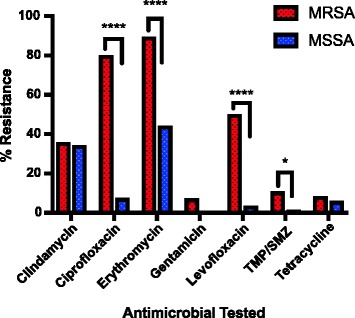
Antimicrobial Resistance Observed in MRSA & MSSA Isolates. Significant differences in antimicrobial resistance between MRSA & MSSA isolates were observed for ciprofloxacin, erythromycin, levofloxacin, and TMP/SMZ. Note: Daptomycin, vancomycin, and linezolid are not included because no resistance was shown for either MRSA or MSSA isolates. Abbreviations: MSSA = methicillin-susceptible *Staphylococcus aureus;* MRSA = methicillin-resistant *Staphylococcus aureus;* TMP/SMZ = trimethoprim/sulfamethoxazole; **** = (*p* < .0001); * = (*p* < .05)

Resistance in MRSA and MSSA was also compared (Fig. 2). As expected, the resistance observed against ciprofloxacin (*p* <0.0001), erythromycin (*p* <0.0001), levofloxacin (*p* <0.0001), and TMP/SMZ (*p* <0.05) was significantly higher for the MRSA isolates compared to the MSSA isolates. This increased resistance is also seen in the MIC_50_/MIC_90_ values, in which MRSA had equal or higher MIC_50_/MIC_90_ values than MSSA in response to ciprofloxacin, erythromycin, levofloxacin, and TMP/SMZ.

### Streptococcus pneumoniae

A total of 30 cultures tested positive for *S. pneumonia*e (Table 3b). Overall, *S. pneumoniae* isolates exhibited relatively low resistance to all of the antimicrobials tested against them. This is further supported by the low MIC_50_ and MIC_90_ values for these antimicrobials. Only erythromycin showed moderate resistance, in which five of the isolates (16.67%) were resistant, as well as a large MIC range from .016 to 32 ug/ml. Vancomycin, clindamycin, and levofloxacin remained effective against *S. pneumoniae,* with each antimicrobial generating over 93% susceptibility.

### Pseudomonas aeruginosa

A total of 42 cultures tested positive for *P. aeruginosa* (Table 4a). The majority of antimicrobials were greater than 90% effective against *P. aeruginosa* with the exception of both meropenem and piperacillin/tazobactam (11.91 and 12.20% resistance respectively). The five isolates that were resistant to meropenem were more likely to be resistant to other classes of antimicrobials (*p* = 0.008), including aminoglycosides and fluoroquinolones.

**Table 4:** Susceptibility profiles of selected Gram-negative species, *P. aeruginosa* (a) and *H. influenzae* (b)

**4a: Susceptibility Profile for 42** ***P. aeruginosa*** **isolates**						
Antimicrobial	MIC (ug/mL)			Interpretation N (%)		
	Range	MIC_50_	MIC_90_	Susceptible	Intermediate	Resistant
Amikacin	≤2 – <16	≤2	16	42 (100)	0 (0)	0 (0)
Ceftazidime	≤1 – 8	4	8	42 (100)	0 (0)	0 (0)
Ciprofloxacin	≤0.25 – ≥4	≤0.25	1	41 (97.6)	0 (0)	1 (2.4)
Cefepime	≤1 – 8	2	<8	42 (100)	0 (0)	0 (0)
Gentamicin	≤1 – ≥16	≤1	4	41 (97.6)	0 (0)	1 (2.4)
Imipenem	≤1 – ≥16	2	4	38 (90.5)	1 (2.4)	3 (7.1)
Levofloxacin	≤0.12 – ≥ 8	1	2	41 (97.6)	0 (0)	1 (2.4)
Meropenem	0.125 – >32	1	8	35 (83.3)	2 (4.8)	5 (11.9)
Piperacillin/Tazobactam	≤4 – ≥128	8	≥128	36 (87.8)	0 (0)	5 (12.2)
Tobramycin*	≤1 – <4	≤1	<4	18 (100)	0 (0)	0 (0)
**4b: Susceptibility Profile for 46** ***H. influenzae*** **isolates**						
Antimicrobial	MIC (ug/mL)			Interpretation N (%)		
	Range	MIC_50_	MIC_90_	Susceptible	Intermediate	Resistant
Ampicillin	0.25 – >256	0.5	>256	22 (66.7)	1 (3)	10 (30.3)
Amoxicillin/Clavulanate	0.5 – 8	1	4	41 (95.3)	0 (0)	2 (4.7)
Ceftriaxone	.004 – 32	0.016	0.125	42 (97.7)	0 (0)	1 (2.3)
Ciprofloxacin*	.008 – 1	0.032	0.5	27 (100)	0 (0)	0 (0)
Levofloxacin	.008 – 2	0.064	0.5	30 (100)	0 (0)	0 (0)
Trimethoprim/Sulfamethoxazole	0.032 – >32	0.5	>32	23 (50)	6 (13)	17 (36.9)

*Antimicrobials that that weren’t tested against 30 or more *P. aeruginosa* and *H. influenzae* isolates

*Abbreviation: MIC* = minimum inhibitory concentration (50 and 90%)

### Haemophilus influenzae

A total of 46 cultures tested positive for *H. influenzae* (Table 4b). The vast majority of isolates were susceptible to most of the antimicrobials tested with the exception of TMP/SMZ and ampicillin, both of which showed high MIC_90_ values. All isolates were tested against TMP/SMZ, with 17 (37%) isolates showing resistance. Of the 33 isolates tested against ampicillin, 10 (30.30%) were resistant.

## Discussion

In this retrospective review, 358 microorganisms were analyzed by a single-center institution over a 6-year study period. A wide variety of Gram-positive and Gram-negative organisms were collected including CoNS, *S. marcescens, K. oxytoca,* and *E. coli,* as well as more common bacterial pathogens such as *S. aureus, S. pneumoniae, H. influenzae,* and *P. aeruginosa.* In this study the number of Gram-negative organisms (49.2%) was nearly equal to the number of Gram-positive organisms (50.8%). While other single-center studies have found a much higher proportion of Gram-positive organisms in collected samples [1, 7, 18, 22–24], this study exhibited a nearly equivalent Gram-positive to Gram-negative ratio. This difference is likely due to the clinical microbiology laboratory protocols that did not require susceptibility testing of CoNS until mid-2015 from eye sources due to the likelihood of these isolates being contaminants. Specifically, there were an additional 152 CoNS isolates, the majority isolated from conjunctival swabs, which did not undergo susceptibility testing. If these isolates had undergone susceptibility testing, the distribution between Gram-positive and Gram-negative bacteria would change considerably (65.5 and 34.5% respectively) and be more in line with previously published studies.

This study also showed an uneven distribution of ocular isolates among age groups. Gram-positive organisms were most common in the elderly, while Gram-negative organisms, except *H. influenzae,* were most common in the middle age ranges. *H. influenzae* isolates had a bimodal distribution, being more common in the youngest and oldest age groups. Additionally MRSA was statistically more likely (*p* < 0.05) to be found in the two oldest age groups (40–64 years and >65 years) when compared to the younger age groups suggesting a higher possibility of MRSA infection with increasing age. While the link between age and risk of infection remains unclear, it may be due to the underdeveloped immune system common in infants and children as well as the depressed immune function observed in the elderly, making the risk of microbial infection in these groups higher [25].

Several drugs are used to treat ocular infections, each with a different mechanism of action. Despite this, many of the pathogens observed in this study are adapting in ways that lead to increased resistance to several classes of antibiotics. Fluoroquinolones, which are a class of drugs often used as first-line treatment for ocular infections, have generally been successful, especially as newer generations have been introduced [1, 22]. However, just like other antimicrobial classes, the systemic use of these fluoroquinolones as first-line broad-spectrum antibiotics tends to lead to increases in resistance due to selective pressure [4, 15, 16, 19]. In this study, 50 and 80% of MRSA isolates showed resistance to levofloxacin and ciprofloxacin respectively, while 3.53 and 7.41% of MSSA isolates showed resistance to levofloxacin and ciprofloxacin, respectively.

Multi-drug resistance (MDR) was observed in many of the MRSA isolates collected in this study (*n* = 12; 42.9%). When compared to MSSA isolates, MRSA isolates have shown statistically significant increases in MDR when compared to MSSA isolates, making them resilient pathogens. However, even with this increased resistance, *S. aureus* isolates have shown limited to no resistance to vancomycin, daptomycin or linezolid, making these treatments reliably effective in bacterial populations that have already shown high resistance to fluoroquinolones, aminoglycosides, and macrolides.

Fluctuating trends in the prevalence of MRSA strains have been reported. Some studies have presented increasing trends in MRSA over extended periods of time [23, 24], while others, including those presented by the CDC, state a decline in MRSA prevalence [26–28]. This study shows a non-statistically significant, decreasing trend over the 6-year study period suggesting that MRSA prevalence has not fluctuated drastically over the last several years. While it is unclear why MRSA prevalence has remained relatively stable in our review, it may result from successful infection control strategies in both hospitals and surrounding communities, or perhaps the cyclical replacement of dominant, infective MRSA strains by MSSA strains over time [27].

In contrast to MRSA, resistance among *S. pneumoniae* and *P. aeruginosa* isolates was low against the antimicrobials tested while resistance among *H. influenzae* isolates was evident primarily against ampicillin and TMP/SMZ. Overall these three organisms did not show alarming resistance to the antimicrobials tested and are consistent with other antimicrobial surveillance studies that have been conducted recently [1, 7, 13, 19].

Several factors are important in the development of antimicrobial resistance in ocular isolates. Overuse of antibiotics is one of the main causes [16]. Other causes include use of an antibiotic when it is not warranted, as in the case of a viral infection, or improper use, such as stopping a course of antibiotics early or using them prophylactically [17]. Since most ocular infections are often resolved through topical application rather than through systemic use, the inherent pharmacokinetic differences between the two must be taken into account when evaluating antimicrobial resistance [29, 30]. Given the high risk of permanent vision loss with eye infection, such as corneal ulcers and endopthalmitis, topical antibiotics tend to be at higher concentrations and are often used as prophylaxis despite limited evidence on their efficacy. The role that topical antibiotics play in antimicrobial resistance is still ambiguous due to the lack of research in this specific area as well as the lack of standardized ocular tissue-specific breakpoints. With the lack of these breakpoints to qualify resistance, CLSI breakpoints have been agreed upon by many researchers as useful indicators of resistance in topical antibiotics and even with the differences mentioned, similar resistance trends are observed in both topical and systemic antibiotic use [31, 32]. Additionally, since systemic antibiotics are still used to treat chronic ocular infections, in such usage, CLSI breakpoints are appropriate. In fact, systemic use of antibiotics may be the key cause of resistance in all isolates, regardless of the source of infection [33, 34]. However, the uncertainty of the role of topical antibiotics is still apparent and therefore requires further attention in order to fully delineate their role.

Limitations of this study include the retrospective nature of this study, which predetermined our sample size, ultimately limiting our analysis of all possible antimicrobial resistance trends. Because there was no standardized protocol for collection of isolates, inevitably there was a large variation in the culturing procedure among physicians. This lack of uniformity may have affected organism recovery in culture. Additionally, the antimicrobial panel underwent small fluctuations from year to year leading to slightly inconsistent susceptibility testing and excluded testing on more recently introduced fluoroquinolones, such as gatifloxacin.

## Conclusion

This 6-year retrospective study reported the numbers of common ocular bacterial isolates and their susceptibility profiles. The study demonstrated a relatively even ratio of Gram-negative to Gram-positive organisms and a variable distribution amongst age groups, depending on the organism. This study also found increased resistance to other drugs among MRSA strains when compared to MSSA strains, as well as moderately low resistance in Gram-negative organisms*.* Future studies should include a panel of commonly used antimicrobials for ocular infections, including more recently introduced fluoroquinolones. To prevent further progression of resistance, findings of this study and other surveillance data on ocular isolates should be used as resources in effective decision making in the treatment of ocular disease and infection.

## Abbreviations

ARMOR: Antibiotic Resistant Monitoring in Ocular Microorganisms
CDC: Center for Disease Control
CLSI: Clinical Laboratory & Standards Institute
CoNS: Coagulase-Negative *Staphylococcus*
MRSA: Methicillin-resistant *S. aureus*
MSSA: Methicillin-Susceptible *S. aureus*
TMP/SMZ: Trimethoprim-Sulfamethoxazole
TRUST: Tracking Resistance in U.S. Today
